# Enhancement of Calcium Chelating Activity in Peptides from Sea Cucumber Ovum through Phosphorylation Modification

**DOI:** 10.3390/foods13121943

**Published:** 2024-06-20

**Authors:** Lingyu Han, Yaoyao Li, Bing Hu, Wei Wang, Jianming Guo, Jixin Yang, Nuo Dong, Yingmei Li, Tingting Li

**Affiliations:** 1 Key Lab of Biotechnology and Bioresources Utilization of Ministry of Education, College of Life Science, Dalian Minzu University, Dalian 116600, China; 20191398@dlnu.edu.cn (L.H.); 18777440369@163.com (Y.L.); hubing19871121@163.com (B.H.); dongnuo931529@163.com (N.D.); 2 NHC Key Laboratory of Food Safety Risk Assessment, China National Center for Food Safety Risk Assessment, Beijing 100022, China; wangweiwsw@cfsa.net.cn; 3 National Center of Technology Innovation for Dairy, Hohhot 010110, China; guojianming@nctid.cn; 4 Faculty of Arts, Science and Technology, Wrexham Glyndwr University, Plas Coch, Mold Road, Wrexham LL11 2AW, UK; j.yang@glyndwr.ac.uk; 5 Linghai Dalian Seafoods Breeding Co., Ltd., Jinzhou 121209, China; hdxixi0112@163.com

**Keywords:** water-soluble peptides, peptide–calcium chelate, phosphorylation modification

## Abstract

Recently, phosphorylation has been applied to peptides to enhance their physiological activity, taking advantage of its modification benefits and the extensive study of functional peptides. In this study, water-soluble peptides (WSPs) of sea cucumber ovum were phosphorylated in order to improve the latter’s calcium binding capacity and calcium absorption. Enzymatic hydrolysis methods were screened via ultraviolet-visible absorption spectroscopy (UV–Vis), the fluorescence spectrum, and calcium chelating ability. Phosphorylated water-soluble peptides (P-WSPs) were characterized via high-performance liquid chromatography, the circular dichroism spectrum, Fourier transform infrared spectroscopy (FTIR), UV–Vis spectroscopy, surface hydrophobicity, and fluorescence spectroscopy. The phosphorus content, calcium chelation rate and absorption rate were investigated. The results demonstrated that phosphorylation enhanced the calcium chelating capacity of WSPs, with the highest capacity reaching 0.96 mmol/L. Phosphate ions caused esterification events, and the carboxyl, amino, and phosphate groups of WSPs and P-WSPs interacted with calcium ions to form these bonds. Calcium-chelated phosphorylated water-soluble peptides (P-WSPs-Ca) demonstrated outstanding stability (calcium retention rates > 80%) in gastrointestinal processes. Our study indicates that these chelates have significant potential to develop into calcium supplements with superior efficacy, bioactivity, and stability.

## 1. Introduction

Sea cucumber, a widely distributed benthic marine creature, is prized in many Asian nations as a wholesome food source [1]. Recently, the aquaculture of sea cucumbers has experienced rapid growth in Asia, particularly in China [2], where the annual production has exceeded 223,700 tons according to the “China Fishery Statistical Yearbook 2022”. However, during industrial processing, sea cucumber ova are frequently discarded as low-value byproducts, which causes environmental concerns. Given the excellent protein content of sea cucumber ova, it is imperative to explore their potential as a value-added material [3]. Peptidomics identified 66 peptide sequences in sea cucumber ovum trypsin hydrolysates, which exhibit exceptional calcium binding capability [4]. We have thus been exploring sea cucumber ovum peptides as a source for achieving exceptionally effective calcium absorption.

Calcium is a critical mineral nutrient in the human body, which is essential for many physiological processes, including nerve transmission, cellular metabolism, muscular relaxation, and immune response [5,6]. Blood pressure, osteoporosis, and osteomalacia are just a few health problems that can be induced by inadequate calcium intake [7]. Currently, calcium supplements available in the market primarily comprise inorganic calcium, organic calcium, and amino acid-derived calcium. However, their poor absorption rate and limited water solubility result in reduced bioavailability in vivo. Due to their exceptional solubility, absorbability, and elevated bioavailability, natural bioactive peptide–calcium chelates have received significant scientific attention [5,8]. These chelates are more stable because of their distinctive structure, created by the joining of minerals to amino acids or peptides [9]. However, the calcium binding potential of most peptide–calcium chelates is rather modest [10]. Therefore, it is crucial to explore techniques to increase peptides’ calcium binding ability without harming their chelates.

According to earlier research, the phosphorylation of peptides is a successful strategy for increasing their relatively poor mineral chelating activity. Phosphorylated peptides can bind to more calcium ions, thereby safeguarding minerals against interactions with dietary components such as phytate and polyphenols, which could otherwise result in their precipitation within the digestive tract. The improved binding increases the bioavailability of calcium. Recent research by Wu et al. [11] showed a significant increase in the calcium chelating rate of phosphorylated porcine collagen peptides. The capacity of herring eggs to chelate calcium increased from 68.81 to 96.82 mg/g after being phosphorylated by sodium trimetaphosphate, according to Sun et al. [12]. The critical reasons for this improvement were hydrogen bonds, hydrophobic contact, and electrostatic interactions between calcium ions and phosphate groups [12]. However, more analysis is necessary to determine whether phosphorylating sea cucumber ovum peptides increases their mineral chelating ability.

In this work, a calcium chelate of phosphorylated sea cucumber ovum peptides was synthesized. To evaluate the effectiveness of phosphorylation in calcium chelation and characterize the products, we employed various techniques, including ultraviolet-visible absorption spectroscopy (UV–Vis), fluorescence spectroscopy, Fourier-transform infrared spectroscopy (FTIR), and circular dichroism (CD). Additionally, we analyzed the calcium binding ability and surface hydrophobicity (H0) of the peptides and phosphorylated products. To further assess chelates’ potential as a brand-new type of calcium supplement for the market, we also examined their stability during simulated in vitro digestion. Our research offers an essential theoretical foundation for creating calcium-chelated phosphorylated water-soluble peptides (P-WSPs-Ca) as a superior calcium supplement.

## 2. Materials and Methods

### 2.1. Materials

Sea cucumber (*Stichopus japonicus*) ova were purchased from Dalian Sea Treasure Breeding Co., Ltd. (Jinzhou, China). Neutral protease (60,000 U/g) and pepsin (1:10,000, ≥250 U/mg) were procured from Solarbio (Shanghai, China), while trypsin (bovine pancreas, 1:250, 250 USPu/mg), Aprotinin hydrochloride (high-purity, 6000 U/mg), glycine (HPLC, ≥99%), and cyanocobalamin (HPLC, ≥99%) were purchased from Yuanye Bio-Technology Co. Ltd. (Shanghai, China). Sodium tripolyphosphate (STPP) and cytochrome C (HPLC, ≥95%) were obtained from Aladdin Reagent Co., Ltd. (Shanghai, China). Gly-Gly-Tyr-Arg (HPLC, ≥95%) was purchased from Yuanye Bio-Technology Co., Ltd. (Shanghai, China). A calcium test kit was purchased from Nanjing Jiancheng Biological Engineering Co., Ltd. (Nanjing, China). A phosphorus (Pi) colorimetric test kit (E-BC-K245-M) was purchased from Thermo Fisher Scientific Co., Ltd. (Shanghai, China). All other chemicals and reagents used in this study were of analytical grade.

### 2.2. Preparation of Sea Cucumber Ovum Proteins

To obtain the water-soluble protein from sea cucumber ovum, 100 g of raw material was mixed with deionized water at a solid–liquid ratio of 1:3 and homogenized for 5 min in a blender. The homogenization buffer was magnetized for 4 h at 4 °C, and the resulting slurry was centrifuged at 10,000× *g* at 4 °C for 10 min. The supernatant was collected, stored in a ziplocked bag, and then freeze-dried. The powder obtained after lyophilization was sea cucumber ovum protein.

### 2.3. Preparation of WSPs

To prepare the water-soluble peptides (WSPs), the water-soluble proteins of sea cucumber ovum were hydrolyzed using a single-enzyme method. An amount of 2 g of sea cucumber ovum powder was added into 100 g of deionized water with stirring until it was fully dissolved. Therefore, the sample solutions was hydrolyzed by neutrase (50 °C, pH 7.0), trypsin (37 °C, pH 8.0), and pepsin (pH 2.0, 37 °C) at a dose of 5000 U g^−1^ protein. After a 5 h reaction, the enzyme was inactivated by heating it to 100 °C for 10 min. The resulting mixture was then centrifuged at 10,000× *g* for 10 min, and the supernatant was freeze-dried and stored at −18 °C until use. The neutral proteases, trypsin, and pepsin were abbreviated as NP, TP, and PP, respectively. The resulting products were marked as WSPs-NP, WSPs-TP, and WSPs-PP.

### 2.4. Phosphorylation of WSPs

The phosphorylated product of WSPs (abbreviated P-WSPs) was prepared via the dry heating method as described by Xiong and Ma [13]. WSPs were dissolved in 0.1 mol/L of sodium tripolyphosphate solution at 20 g/L. The pHs of the mixed solutions were adjusted to 3.0, 4.0, 5.0, 6.0, 7.0, 8.0, and 9.0 using 1 M HCl and 1 M NaOH, and then lyophilized in a freeze dryer (FD-1A-50+, BIOCOOL, Beijing, China). The resulting dry powder was incubated at 45 °C and 79% relative humidity in an SPX-150B-Z incubator (Shanghai, China) for 12 h. The products obtained at varying pH values were dissolved in deionized water and dialyzed for 48 h to remove any unbound phosphate ions. The phosphorylated products of WSPs were named P-WSPs-3, P-WSPs-4, P-WSPs-5, P-WSPs-6, P-WSPs-7, P-WSPs-8, and P-WSPs-9, according to the pH values used to prepare them, and were stored in a sealed container at −20 °C for later use.

The phosphorus content was determined using a phosphorylation assay kit (E-BC-K245-M, Elabscience Biotechnology Co., Ltd., Wuhan, China). Normal saline with a mass concentration of 0.9% was used to dilute the phosphorus standard, and the concentration was 0, 0.2, 0.5, 0.8, 1.0, 1.5, and 2.0 mmol/L. Taking the standard concentration (mmol/L) as the X axis and the difference between the absorbance of different concentrations of standard phosphorus solution at 660 nm minus the absorbance of blank group (absorbance when the standard concentration is 0) as the Y axis, a standard curve was drawn to obtain the equation. The standard curve equation was Y = 0.9795X + 0.0233; R^2^ = 0.9972. The sample was dissolved in deionized water at a dilution concentration of 1 mg/mL. A 100 μL sample was placed into a 1.5 mL EP tube, and reagent 4 was added, thoroughly mixed, and centrifuged at 1100× *g* for 10 min. The absorbance was measured at 660 nm by taking a 35 μL supernatant and adding 200 μL of color developer into 96-well plate. The formula for calculating the degree of phosphorylation is as follows: Pi (mmol L) = (∆A_660_ − b) ÷ a × 5 × f (∆A_660_, sample absorbance minus blank absorbance; b, curve slope; f, the dilution ratio before the sample is added to the detection system).

### 2.5. Preparation of WSPs-Ca and P-WSPs-Ca

WSPs and P-WSPs samples were dissolved in deionized water to a final concentration of 50 mg/mL. Then, CaCl_2_ was added with a specified peptide/CaCl_2_ ratio (*w/w*) of 3:1 to form calcium chelates of these compounds. The solution was then stirred at 50 °C for 20 min. To eliminate any free calcium ions, ethanol was then added to the reactant to a final concentration of 85%. The resulting mixture was centrifuged at 13,805× *g* and 4 °C for 5 min, and the precipitate was collected and then lyophilized at −38 °C for 24 h. The calcium chelates of WSPs and P-WSPs were designated as WSPs-Ca and P-WSPs-Ca, respectively.

### 2.6. Calcium Binding Capacity Assay

To test the calcium binding abilities of WSPs and P-WSPs, these peptides were dissolved in a phosphate-buffered solution (PBS, 0.2 M, pH 7) with a concentration of 0.5 mg/mL. Next, 5 mM CaCl_2_ was added to the solution, which was then stirred at 37 °C for 1 h while the pH was maintained at 7.0. After centrifugation at 6900× *g* for 5 min, the supernatant was collected, and the calcium content was measured via the microporous method using a calcium assay kit (C004-2-1, Nanjing Jiancheng Bioengineering Institute, Nanjing, China).

### 2.7. Structural Characterization

#### 2.7.1. Molecular Weight Distribution

The molecular weight (MW) distribution profiles of WSPs-NP were determined using gel permeation chromatography with an Xtimate SEC-120 column (300 × 7.8 mm, 5 μm) based on an Elite P230 high-performance liquid chromatography (HPLC) system, in accordance with the method of Sun et al. [3], with some modifications. Briefly, 1 mg/mL solutions of both WSPs and P-WSPs, filtered through a 0.22 μm filter, were prepared, and 40 µL of each solution was loaded into the HPLC column. The mobile phase used was acetonitrile/water/trifluoroacetic acid in a ratio of 30:70:0.1 (*v/v/v*). The sample was eluted at a 0.5 mL/min flow rate and monitored at 220 nm using a UV detector. Standards of varying molecular weights, which included glycine (75 Da), Gly-Gly-Tyr-Arg (307 Da), cyanocobalamin (1355 Da), aprotinin hydrochloride (6511.51 Da), and cytochrome C (12,500 Da), were used for calibration. The equation below shows the standard calibration curve obtained (Supplementary data Figure S1).

$$Y=-0.2022X+6.5916\;(R^{2}=0.9941)$$

where Y denotes the logarithm of the relative molecular weight of samples (lg MW), and X denotes the corresponding retention time.

#### 2.7.2. Circular Dichroism (CD) Spectrum

The CD spectra were obtained following the method described by Sun et al. [12], with minor modifications, using a 4710KL-04W-B10 CD spectropolarimeter (Shanghai, China). The far-UV CD spectra of 1 mg/mL of WSPs and P-WSPs in 50 mM phosphate buffer (pH 7.4) were recorded at 25 °C, with a spectral resolution of 0.1 nm, a scan speed of 100 nm/s, a response time of 0.125 s, and a bandwidth of 1 nm, in the region of 190 to 260 nm. The optical path of 0.1 cm quartz cells was utilized. The acquisition involved gathering 16 scans in total, which were then averaged. An average from three different spectra was used for each sample.

#### 2.7.3. Fourier Transform Infrared Spectroscopy (FTIR)

The samples were lyophilized, combined with dry potassium bromide powder, homogenized, and crushed into pellets using a tableting mode to measure the IR spectra. Fourier transform infrared (FTIR) spectroscopy was utilized to perform the analysis, with a wavenumber range of 400–4000 cm^−1^, using an IRPrestige21 spectrometer (Shimadzu, Japan). The equipment had a resolution of 2.0 cm^−1^, and the air background was taken prior to the measurement of each sample.

#### 2.7.4. Ultraviolet-Visible Absorption Spectroscopy (UV–Vis)

WSPs and P-WSPs were dissolved in water at a concentration of 0.1 mg/mL, and a 1000 mmol/L CaCl_2_ solution was added to the solution (with a volume ratio of 0, 20:1, 10:1, 2:1, and 1:1). Peptide–calcium complexes were prepared by incubating the sample solutions at 50 °C at pH 8.5 for 1 h. The lyophilized materials (WSPs and P-WSPs) were dissolved in PBS (20 mM, pH 7.4) to a final concentration of 1 mg/mL for analysis. A UV-Vis spectrophotometer (UV-2450, Shimadzu, Japan) with a 200–480 nm wavelength range was used to record the UV spectrum of each solution. Briefly, 20 mM PBS was used as a blank control for all the measurements.

#### 2.7.5. Determination of Surface Hydrophobicity (H_0_)

The fluorescent probe ANS (8-anilino-1-naphthalenesulfonic acid ammonium salt) was used, in accordance with the modified approach of Luo et al. [14], to measure the H_0_, which stands for the hydrophobicity of proteins. WSPs-NP and P-WSPs were diluted to protein concentrations of 0.0125, 0.025, 0.05, 0.1, and 0.2 mg/mL in phosphate buffer (20 mM, pH 7) and then combined with an 8 mM ANS solution at a volume ratio of 200:1 in a separate container. The mixture’s fluorescence intensity was measured at 370/470 nm (excitation/emission). The initial slope of the linear regression between the protein concentration (mg/mL) and fluorescence intensity was computed to determine the H0. ANS can be used as an accurate method of measuring H_0_ because of its strong affinity for binding to proteins’ hydrophobic surfaces.

#### 2.7.6. Fluorescence Spectroscopy

The fluorescence spectra of WSPs, P-WSPs, and their phosphorylated products were also recorded using the Perkin Elmer (USA LS-55) luminescence spectrometer [15]. Peptide–calcium complexes were prepared in the same manner as described in the UV-Vis analysis above. Samples were dissolved in phosphate buffer (20 mM, pH 7.4) at a 0.5 mg/mL concentration. The emission spectra were examined between 280 and 500 nm with a slit width of 5 nm, and the excitation wavelength was set at 275 nm.

### 2.8. Simulated Digestion In Vitro

With a few minor adjustments, the procedures of O’Loughlin et al. [16] were used to imitate the stomach digestion of the materials. An amount of 40 mg of pepsin was dissolved in 1 mL of 0.1 mol/L HCl to prepare a simulated gastric fluid. Then, 2 mg trypsin and 12 mg bile salt were dissolved in 1 mL of 0.1 mol/L NaHCO_3_ to prepare a simulated intestinal digestive fluid. Deionized water was used to dissolve WSPs-NP-Ca and P-WSPs-Ca to a concentration of 1 mg/mL, 1 M HCl was used to bring the pH of the sample solution down to 2.0, and it was then incubated at 37 °C for 30 min. Simulated gastric fluid was added at a 1:100 (*w/w*) enzyme/substrate ratio, and incubation was continued at 37 °C for 120 min. Next, 1 M NaOH was used to adjust the pH to 7 and simulated intestinal fluid was added at an enzyme/substrate ratio of 1:50 (*w/w*), which was further incubated for 4 h, with sampling intervals of 1 h. Then, the fluid was immediately boiled in a water bath for 10 min to terminate the digestion process. The resultant solutions were centrifuged at 10,000× *g* for 10 min. After collecting the supernatants, their calcium content was assessed using a calcium assay kit and the microporous method.

### 2.9. Statistical Analysis

The experiments were conducted in triplicate, and the outcome was expressed as the mean ± standard deviation. Data recording and calculation were performed using Excel (2019), while SPSS 22.0 was used for statistical analysis. Duncan’s multiple-range test was performed to determine the statistical differences between the samples, with a *p*-value of 0.05 as the significance threshold. One-way analysis of variance (ANOVA) was performed to evaluate the differences between groups.

## 3. Results and Discussion

### 3.1. The Calcium Binding Capacity of WSPs

**Figure 1:**
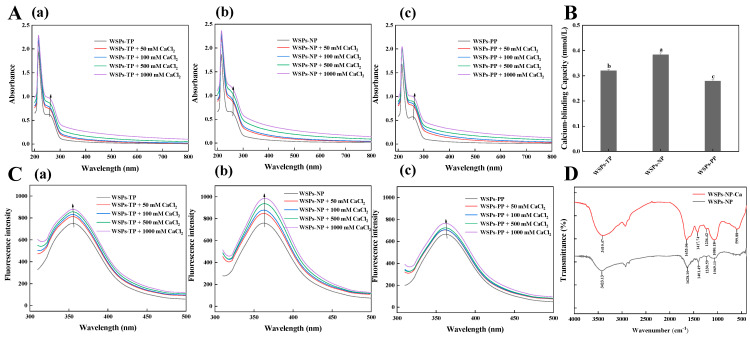
(**A**) UV−Vis spectra of (**a**) WSPs−TP, (**b**) WSPs−NP, and (**c**) WSPs−PP with different concentrations of CaCl_2_ over a wavelength range from 200 to 800 nm. (**B**) Calcium binding capacity of WSPs−TP, WSPs−NP, and WSPs−PP. (**C**) Fluorescence spectra of (**a**) WSPs−TP, (**b**) WSPs−NP, and (**c**) WSPs−PP with different concentrations of CaCl_2_ over an emission wavelength ranging from 310 to 500 nm at an excitation wavelength of 295 nm. (**D**) FTIR spectra of WSPs−NP and WSPs−NP−Ca in the regions from 4000 to 400 cm^−1^. Note: The different letters mean significant differences *p* < 0.05 within the same indicator.

The calcium chelating activity of WSPs was evaluated using UV-Vis, fluorescence spectroscopy, and FTIR. The UV-Vis spectra (Figure 1A) show the ability of three peptides to interact with calcium. Maximum absorption occurred at approximately 220 nm. These unique absorption patterns can be attributed to the n → π* transition of C=O in the peptides [12]. After the addition of calcium ions, the maximum absorption of peptides was enhanced and redshifted because the metal ions and their partially formed ligands absorbed ultraviolet wavelengths and formed electronic transitions. As the valence electron transitions of corresponding atoms changed during the chelation process, the absorption of ultraviolet light between calcium ions and peptides also changed [12]. As the CaCl_2_ concentration increased, the absorption band intensity increased and shifted. Moreover, compared with the spectrum of WSPs, WSPs-Ca exhibited a gentle absorption peak in the range of 250–260 nm, corresponding to the absorption peak of phenylalanine [12,17]. Similar absorption intensity increases and redshifts in the maximum absorption have been observed in herring egg phosphopeptide–calcium complexes [12], demonstrating how calcium ion binding affects the spatial structure of peptides’ chiral chromospheres (C=O and –COOH) and auxochromes (–OH and –NH_2_) [18]. These findings imply that the WSPs of sea cucumber ova can combine with calcium to produce a novel chemical compound.

The order of calcium chelating ability was NP > TP > PP (Figure 1B). The calcium chelating capacity of the peptides after 5 h of hydrolysis with neutral protease was 0.38 mmol/L. Other relevant studies have reported higher calcium binding capacities for hydrolysates produced with trypsin [17]. For instance, Wu et al. [17] hydrolyzed pig bones sequentially using Flavourzyme, alcalase, neutrase, pepsin, and trypsin to produce a collagen peptide with outstanding calcium binding abilities. Due to unique cleavage locations, the type of protease utilized affects the hydrolysates’ calcium binding ability [19]. Therefore, WSPs-NP were preferable for the preparation of phosphorylated products.

Figure 1C demonstrates that the fluorescence emission peak of WSPs at 360 nm may have been caused by tryptophan [12]. Increasing CaCl_2_ from 0 to 1000 mmol/L led to a distinct increase in WSPs’ fluorescence intensity. The highest fluorescence intensity was recorded at a CaCl_2_ concentration of 1000 mmol/L, which suggests that amino acid or polypeptide structures can undergo folding and aggregation upon chelation with calcium ions. Sun et al. [12] reported similar results, where an increase in CaCl_2_ concentration led to a rise in herring egg phosphopeptides’ endogenous fluorescence, accompanied by a redshift. Peptide folding and calcium ion-induced fluorescence quenching, which impact calcium binding peptides, can alter peptide structure and reduce fluorescence intensity [18].

Figure 1D displays the FTIR spectra of WSPs and WSPs-Ca. The shifts in absorption peaks observed in the FTIR spectra show the potential binding sites of mineral ions and amino acids within peptides. O–H and N–H stretching vibrations were principally responsible for the WSPs-NP absorption peak at 3423.13 cm^−1^. This signal changed to 3410.47 cm^−1^ after binding to calcium ions, indicating that O–H and N–H had contributed to chelate formation [5]. The carboxylic group is involved in covalent bonding to the metal ion, as evidenced by changes in the C=O stretching vibration in the region 1655–1620 cm^−1^. C–O stretching and –OH deformation vibrations were located at 1100–1000 cm^−1^, with a wavenumber of 1069 cm^−1^ shifting to a higher frequency of 1080 cm^−1^. The peak (1401.49 cm^−1^) for the –COO– carboxylate group moved to a higher frequency (1417.74 cm^−1^), indicating that –COOH was likely bound to the calcium ion and converted into –COO-Ca. According to the infrared spectra of WSPs and WSPs-Ca, COO–, N–H, O–H, and C=O groups may have contributed to the production of WSPs-Ca.

### 3.2. Structural Characterization of WSPs-NP and P-WSPs

#### 3.2.1. Molecular Weight Distribution

**Figure 2:**
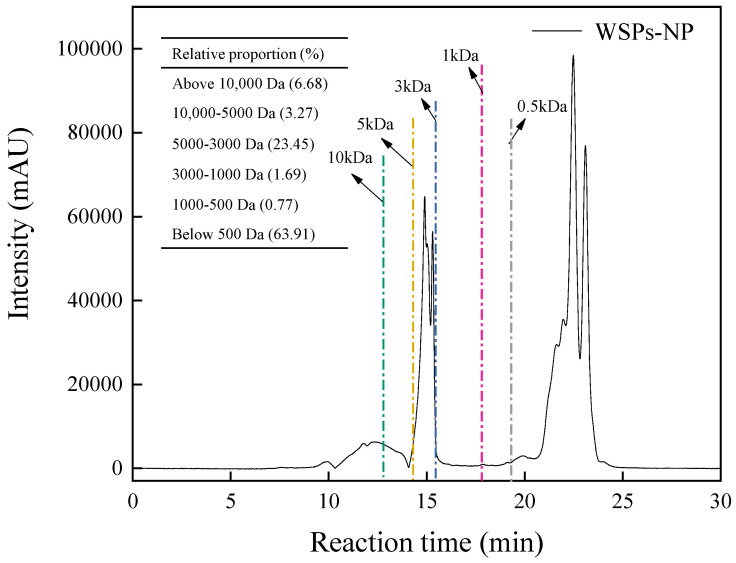
The molecular mass (MW) distribution of WSPs−NP.

Peptides may be identified by their molecular weight, which significantly impacts biological activity and intestinal calcium absorption. Gel permeation chromatography was used to measure the molecular weight distribution of WSPs-NP, following the method by Qi et al. [20]. Figure 2 displays the MW distribution profiles; the relative contents of peptides with MWs of 500 Da or less, 500–1000 Da, 1000–3000 Da, and 3000–5000 Da were 63.91%, 0.77%, 1.69% and 23.45%, respectively, suggesting that most peptides in WSPs-NP were less than 5000 Da. Various studies demonstrate that low-molecular-weight peptides exhibit significant absorption properties and are biologically active in binding to receptor proteins [3,17,20]. Fish skin collagen peptides with calcium chelating activity have a molecular weight distribution mostly between 180 and 2000 Da [21]. In addition, Torres-Fuentes et al. [22] discovered that smaller peptides (below 500 Da) generated from chickpea protein had greater iron binding ability than larger peptides (above 500 Da). These results imply that the calcium binding ability of WSPs-NP is significantly influenced by their molecular mass and that low-molecular-weight hydrolysates are advantageous for calcium binding.

#### 3.2.2. CD Spectral Analysis

**Figure 3:**
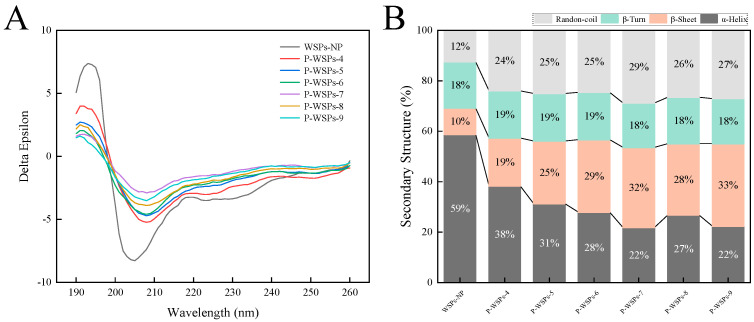
(**A**) The circular dichroism (CD) spectrum and (**B**) secondary structure content of WSPs−NP and P−WSPs.

The far-UV CD spectra of WSPs-NP and P-WSPs are depicted in Figure 3A. Circular dichroism is an established method for studying the secondary structure of proteins or peptides in the “far-ultraviolet” spectral region (190–260 nm) [12].CD spectra analysis showed that the secondary structure of P-WSPs consisted of four distinct conformations, including helixes, sheets, and turns, with a preference for random coil shapes. The negative minimum at around 209 nm and the shoulder at about 222 nm indicate the presence of an α-helical structure [15]. A β-sheet had an intense peak at 195 nm and a negative minimum at about 217 nm [15]. While the far-UV CD spectra patterns of WSPs and P-WSPs revealed some similarities, WSPs showed notable deviations from P-WSPs in these results.

CDNN 2.1 software was used to examine the information contained in secondary structures. α-helix content in P-WSPs decreased, while β-sheet structures significantly increased. Phosphate groups were found to induce a structural transformation of WSPs-NP from α-helixes to β-sheets. As a result, α-helix and β-sheet content accounted for 69% of the secondary structure in WSPs-NP, a highly ordered and stable protein. The β-turn structure of P-WSPs did not change significantly. These results suggest that phosphate groups affect the hydrogen bonds that maintain the protein’s secondary structure (such as the hydrogen bonds between the hydrogen atoms of the amide group and the oxygen atoms of the carbonyl group), resulting in the interconversion of the secondary structures [23]. The random structure content rose from 12% at 0 h to 25% after 12 h of phosphorylation. Overall, compared with WSPs-NP, P-WSPs were more flexible and disordered overall, despite each component of the secondary structure having a varied tendency to change.

#### 3.2.3. FTIR and Phosphorus Content

**Figure 4:**
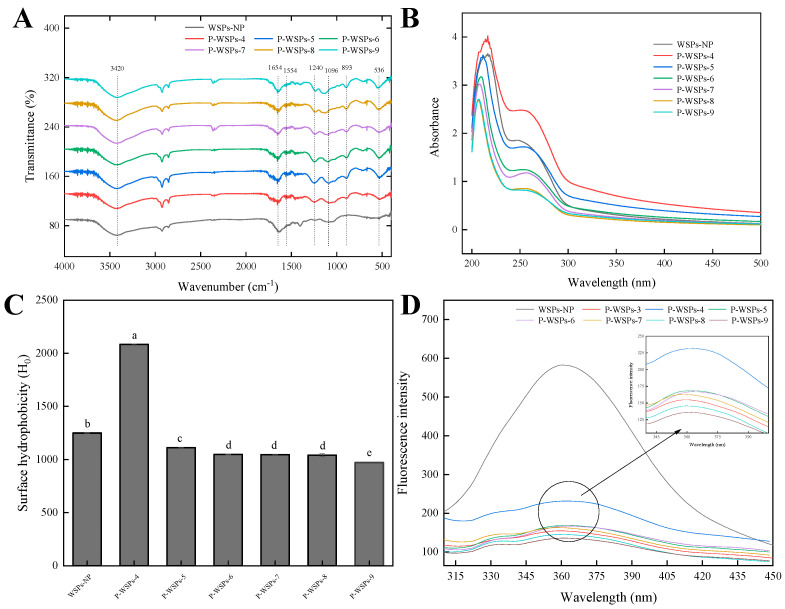
(**A**) FTIR spectra of WSPs−NP and P-WSPs in the regions from 4000 to 400 cm^−1^. (**B**) UV−Vis spectra of WSPs−NP and P−WSPs over a wavelength range from 200 to 480 nm. (**C**) Surface hydrophobicity index of WSPs−NP and P−WSPs. The data shown are the averages of triplicate independent experiments. Bars denoted with different lowercase letters are significantly different (*p* < 0.05). (**D**) The fluorescence spectra of WSPs−NP and P−WSPs over an emission wavelength ranging from 280 to 500 nm at an excitation wavelength of 275 nm.

Figure 4A displays the FTIR spectra of WSPs-NP and P-WSPs. Amide A exhibits a distinct absorption peak at 3420 cm^−1^, which is primarily related to the stretching vibration of O–H and N–H [24]. The most easily recognized protein structures were deduced from two prominent peaks at around 1654 cm^−1^ and 1554 cm^−1^, corresponding to amides I and II, respectively [15]. After phosphorylation, the absorption peaks in WSPs rose at 1240 cm^−1^, most likely due to C–N stretching vibrations and N–H deformation from amide bonds [24]. Phosphorylated WSPs had two distinct peaks at around 1096 and 893 cm^−1^, indicative of P=O and P–O stretching vibrations, respectively, which were absent in the absorption spectra of unphosphorylated WSPs. These features confirmed that sodium tripolyphosphate was successfully linked to WSPs, consistent with previous reports [24,25].

The results in Table 1 show that the phosphorus content of P-WSPs increased with increasing pH, with a maximum value of 3.24 mmol/L at pH = 9.0. This result agrees with earlier research in which an increase in pH increased the phosphorus content of pig bone collagen peptides [11]. Under neutral or acidic circumstances, STPP is unstable and readily converts into sodium pyrophosphate, which has lower activity and reduced phosphorylation. The differential phosphorus content observed may be attributed to differences in the phosphorylation sites and WSPs-NP locations at varying pH values. Previous research found that most phosphorylation sites were located on serine residues after ovalbumin phosphorylation using dry heating at three pH levels [13]. Nevertheless, only a minor portion of the phosphorylation process occurred in threonine residues. Similarly, Wang et al. [26] reported that dry heating ovalbumin with pyrophosphate for 5 days resulted in phosphorylation solely on serine residues. Furthermore, studies have shown that phosphate groups can attach to oxygen in seryl, threonyl, aspartyl (β-carboxyl), and tyrosyl residues, or to nitrogen in lysyl (ε-amino) and histidyl (1 and 3) residues [27].

#### 3.2.4. UV-Vis Spectra

Figure 4B displays the UV–Vis absorption spectra of WSPs-NP and P-WSPs. Variations in the peptide bonds present might affect their absorption peak, which generally occurs at about 210 nm [28]. Moreover, characteristic absorption peaks around 260 nm were observed, which align with the spectral characteristics of aromatic amino acid residues in the peptide [12]. Phosphorylation changed the peak intensity corresponding to λ = 210 nm and λ = 260 nm. Notably, the products phosphorylated at pH 4 exhibited high surface hydrophobicity and UV absorption due to exposure to tryptophan and tyrosine hydrophobic groups, compared with WSPs-NP. Conversely, those obtained at other pH values were more likely embedded within protein molecules and exhibited lower absorption at 260 nm than WSPs-NP did. Due to the esterification process and a network structure forming between sea cucumber ovum peptides and phosphate ions, phosphatylation alteration reduced amino acid residue exposure and lowered its UV absorbance.

#### 3.2.5. H_0_ Analysis

Figure 4C presents the H_0_ indices of phosphorylated peptides obtained at different pH values. The trend of the H_0_ changes is opposite to the trend of the UV absorption peak intensity (Figure 4B). Compared with sea cucumber ovum peptides, the H_0_ value of P-WSPs-4 significantly increased. Conversely, the H_0_ values of other phosphorylated sea cucumber ovum peptides (P-WSPs-5, P-WSPs-6, P-WSPs-7, P-WSPs-8, and P-WSPs-9) decreased. These experimental H_0_ results agreed with the UV-Vis data. The reason is the exposure of tryptophan and tyrosine hydrophobic groups within P-WSPs-4 particles, which reduced UV absorption and increased surface hydrophobicity. The findings align with previous research results, which demonstrated that phosphorylation can increase the hydrophobicity of protein residues, in contrast to their unphosphorylated counterparts [28]. In addition to heat’s impact, the partial or total unfolding and denaturation of the protein chain may result from STPP binding to WSPs-NP molecule chains. As a result, hydrophobic amino acids previously buried within the protein’s inner region may become exposed. Further increasing the pH of phosphorylation decreased the H_0_ of the phosphorylated product due to a higher amount of nonpolar amino acids being present in the core of the globular protein molecule, with polar amino acids distributed on the molecule’s surface [12].

#### 3.2.6. Fluorescence Spectroscopy

As depicted in Figure 4D, the maximum fluorescence intensity of both peptides and their phosphorylated products was observed at a 360 nm wavelength upon excitation at 295 nm. The observed trend in fluorescence intensity changes was consistent with the phosphorus content. Additionally, it was apparent that the fluorescence intensity of P-WSPs was lower than that of WSPs-NP, consistent with previous reports [28]. The reduced fluorescence intensity may have been due to modifications in protein structure, resulting in the chromophore being exposed to a more polar environment [29]. Additionally, the phosphorylation-induced conformational modifications enhanced the exposure of tryptophan (Trp) residues, which resulted in a modest fluorescence blue shift. This suggests a structural difference between phosphorylated and unphosphorylated peptides [28].

### 3.3. The Calcium Binding Capacity of P-WSPs

Table 1 shows that the calcium binding capacity of unphosphorylated WSPs was 0.38 mmol/L. However, phosphorylation by STPP significantly (*p* < 0.05) increased the calcium binding ability of WSPs to 0.96 mmol/L (P-WSPs-4), consistent with previous findings that demonstrated higher binding constants of phosphorylated peptides compared with those of their dephosphorylated counterparts [12]. Notably, the calcium chelating activity of phosphorylated WSPs was opposite to the trend of the phosphorylation degree. As the degree of phosphorylation increased, steric hindrances were strengthened, which may have adversely affected the chelation reaction of P-WSPs with carboxyl and amino groups. This phenomenon may also be related to the phosphorylation site of the product. Zong et al. [30] found that phosphopeptides containing more Ser(P)s inhibit calcium phosphate precipitation. In addition, phosphopeptides with discontinuous Ser(P)s have superior calcium binding characteristics compared with those with continuous Ser(P)s. Ser(P) in phosphopeptides is therefore essential for avoiding calcium phosphate precipitation and controlling its quantity and organization.

**Figure 5:**
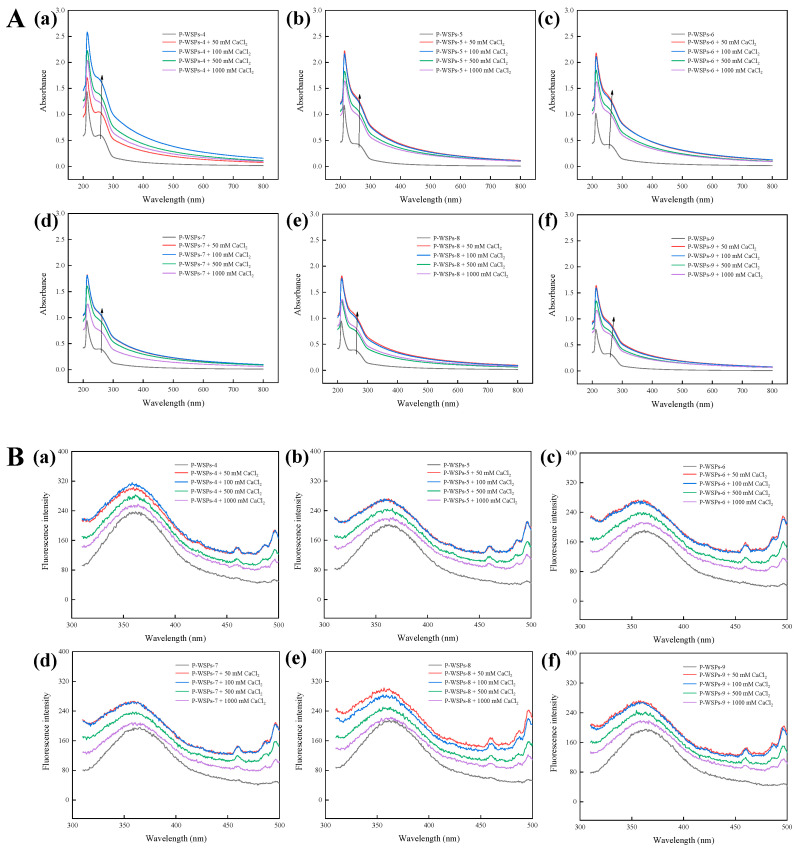
(**A**) UV−Vis spectra of P−WSPs with different concentrations (50 mM, 100 mM, 500 mM, and 1000 mM) of CaCl_2_ over a wavelength range from 200 to 800 nm, (**a**) P−WSPs−4 (**b**) P−WSPs−5 (**c**) P−WSPs−6 (**d**) P−WSPs−7 (**e**) P−WSPs−8 (**f**) P−WSPs−9; (**B**) Fluorescence spectra of P−WSPs with different concentrations (50 mM, 100 mM, 500 mM, and 1000 mM) of CaCl_2_ over a emission wavelength ranging from 310 to 500 nm at an excitation wavelength of 295 nm, (**a**) P−WSPs−4 (**b**) P−WSPs−5 (**c**) P−WSPs−6 (**d**) P−WSPs−7 (**e**) P−WSPs−8 (**f**) P−WSPs−9.

Compared with P-WSPs, the UV-Vis spectra of its calcium chelate show a significant change (Figure 5A), with a maximum absorption peak at 210 nm, which typically correlates to the peptides’ unique carbonyl groups, carboxyl groups, and amide bonds [31]. The absorbance of aromatic amino acid residues is attributed to a second absorption peak at 260 nm. However, the strength of this peak initially increased and subsequently declined while moving towards the red region when the CaCl_2_ concentration was raised. The observed increase in absorption intensity, as reported by Yang et al. [32], may be attributed to alterations in the chiral spatial structure of P-WSPs upon binding with calcium cations, affecting the carbonyl and carboxyl groups as well as chromophores such as hydroxyl and amino [31]. Wang et al. [33] observed that upon combining casein phosphopeptide with calcium, the UV absorption peak intensity reduced and shifted towards shorter wavelengths. This can be attributed to metal ions and ligands absorbing ultraviolet wavelengths and undergoing electronic transitions, as well as the resulting transition and dissociation of electrons caused by the bonding between chelate ions and ligands. In addition, the transition of valence electrons in corresponding atoms changes during this process, altering the absorption of ultraviolet light during chelation. These results confirm the successful binding of P-WSPs to calcium. Moreover, P-WSPs demonstrated a more robust interaction with calcium, as evidenced by a superior phenylalanine transition at 250–260 nm compared with that of unphosphorylated sea cucumber ovum peptides.

Fluorescence produced by tryptophan has an emission peak range of about 350 nm when the excitation wavelength is 295 nm. As the Ca concentration increased, the fluorescence intensity at 360 nm rose and then decreased (Figure 5B), demonstrating that adding calcium ions and phosphate groups caused structural modifications in WSPs-NP. The intensity drop can be attributable to peptides with aromatic amino acids dimming their fluorescence after interacting with calcium ions. Cai et al. [34] found that the endogenous fluorescence intensity of a dipeptide (Phe-Tyr) decreased with increasing calcium ion concentration and remained unchanged after it reached 5 mM. The complicated interaction between chromophores and Ca^2+^ may alter fluorescence intensity due to energy shifts in the excited state [18]. The transfer of amino acids to the molecular surface due to metal ions can also change the highest absorption peak [17].

### 3.4. In Vitro Simulated Digestion

**Figure 6:**
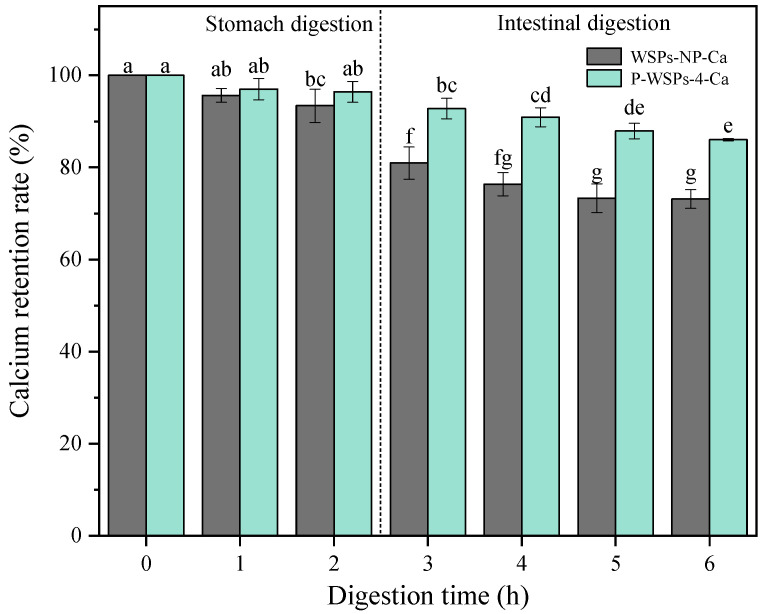
Stability of peptide–calcium chelates of WSPs and P-WSPs-4 under simulated gastrointestinal digestion. Annotation: The different letters mean significant differences *p* < 0.05 within the same indicator.

The calcium retention rate of peptide–calcium chelates in the gastrointestinal enzyme environment is illustrated in Figure 6. After 2 h of pepsin digestion and 4 h of trypsin digestion, the calcium retention rates of WSPs-NP were 93.39% and 73.3%, respectively, while those of P-WSPs were 96.41% and 86.02%, respectively. The outcomes showed that pepsin had a negligible impact on the chelate’s stability. Luo et al. [35] discussed the calcium retention rate of Ca-P-CP at different pH values and found that the chelate was pH-sensitive, and its instability in acidic environments caused the bound calcium to dissociate into an ionic form. Meanwhile, in the digestive system, which used two proteases provided sequentially at the appropriate pHs, trypsin may have degraded the peptides, ultimately decreasing their calcium binding ability [17]. Yang et al. [32] determined the molecular weight distribution of low-molecular-weight collagen peptide calcium chelate (CP_LW_-Ca) during simulated digestion, and found that enzymatic hydrolysis had no significant effect on CP_LW_-Ca during gastric digestion. After entering the intestinal digestive system, the molecular weight of CP_LW_-Ca decreased gradually with the extension of digestion time. Combined with the calcium retention rate of CPLW-Ca in the simulated digestion process, the results showed that under the action of trypsin, the high-molecular-weight calcium binding peptides could be transformed into low-molecular-weight peptides, thus reducing the binding stability of calcium ions. Phosphorylated peptides had significantly improved bioavailability compared with peptides. Peptides that combine with a phosphate group can hinder the formation of insoluble calcium phosphate in the intestine, consequently enhancing calcium’s bioavailability. Due to their high stability and calcium retention ability during chelation and digestion processes, P-WSPs-4 can aid in calcium absorption.

## 4. Conclusions

The calcium retention rate of peptide–calcium chelates in the gastrointestinal enzyme environment is illustrated in Figure 6. After 2 h of pepsin digestion and 4 h of trypsin digestion, the calcium retention rates of WSPs-NP were 93.39% and 73.3%, respectively, while those of P-WSPs were 96.41% and 86.02%, respectively. The outcomes showed that pepsin had a negligible impact on the chelate’s stability. The chelate was discovered to be pH-sensitive, and its instability in acidic environments caused the bound calcium to dissociate into an ionic form. In the digestive system, which used two proteases provided sequentially at the appropriate pHs, trypsin may have degraded the peptides, ultimately decreasing their calcium binding ability [17]. Phosphorylated peptides had significantly improved bioavailability compared with peptides. Their potential interaction with bile acids of the in vitro gastrointestinal digestion system may have decreased calcium chelation. Calcium has low bioavailability because it tends to react with phytic acid or oxalic acid in the stomach, forming insoluble precipitates or Ca(OH)_2_ in the small intestine [17,34]. Trypsin may reduce the peptide’s ability to bind calcium during intestinal digestion. Polypeptides that combine with phosphate can hinder the formation of insoluble calcium phosphate in the intestine, consequently enhancing calcium’s bioavailability. Due to their high stability and calcium retention ability during chelation and digestion processes, P-WSPs-4 can aid in calcium absorption.

## Figures and Tables

**Table 1:** Degree of phosphorylation modification and calcium blinding capacity of WSPs-NP and P-WSPs.

Sample	Phosphorus Content (mmol/L)	Calcium Binding Capacity (mmol/L)
WSPs-NP	0.43 ± 0.04	0.38 ± 0.01
P-WSPs-3	2.13 ± 0.03	0.58 ± 0.01
P-WSPs-4	0.96 ± 0.09	0.96 ± 0.01
P-WSPs-5	1.61 ± 0.02	0.66 ± 0.03
P-WSPs-6	1.82 ± 0.06	0.63 ± 0.04
P-WSPs-7	2.04 ± 0.04	0.59 ± 0.03
P-WSPs-8	2.24 ± 0.03	0.58 ± 0.02
P-WSPs-9	2.97 ± 0.15	0.54 ± 0.02

## Data Availability

The original contributions presented in the study are included in the article/Supplementary Materials, further inquiries can be directed to the corresponding author.

## Supplementary Materials

The following supporting information can be downloaded at: https://www.mdpi.com/article/10.3390/foods13121943/s1, Figure S1. HPLC diagram of bovine of standard substance. (A) Glycine, Gly-Gly-Tyr-Arg, aprotinin hydrochloride and cytochrome C; (B) cyanocobalamin; (C) Standard curve of five standard substance. Figure S2. Standard curve of phosphorus standard solutions.
