# Supplementary material for: Enhancement of Calcium Chelating Activity in Peptides from Sea Cucumber Ovum through Phosphorylation Modification
Source: Foods. 2024 Jun 20;13(12):1943. doi: 10.3390/foods13121943 (PMC11202592; doi:10.3390/foods13121943)
Supplement: Supplementary file 1 [file foods-13-01943-s001.zip › foods-3032290-supplementary.pdf]

List of Supplementary data Figures

Supplementary data Figure S1. HPLC diagram of bovine of standard substance. (A) Glycine, Gly-Gly-Tyr-Arg, aprotinin hydrochloride and cytochrome C; (B) cyanocobalamin; (C) Standard curve of five standard substance

Supplementary data Figure S2. Standard curve of phosphorus standard solutions

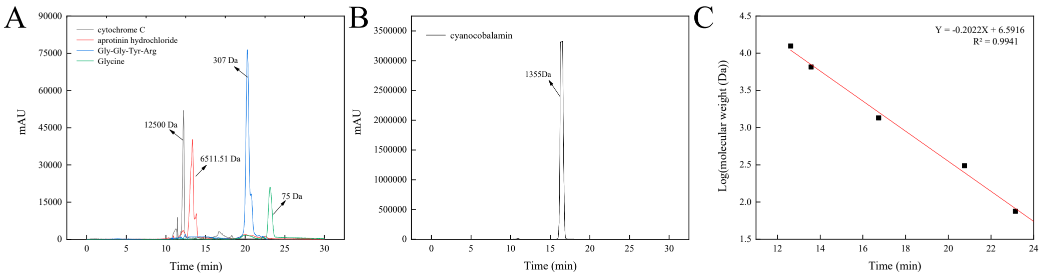

Figure S1. HPLC diagram of bovine of standard substance. (A) Glycine, Gly-Gly-Tyr-Arg, aprotinin hydrochloride and cytochrome C; (B) cyanocobalamin; (C) Standard curve of five standard substance

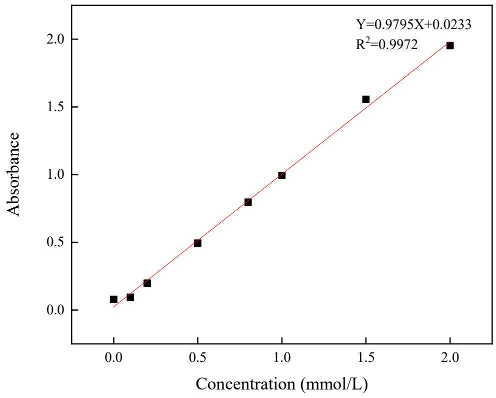

Figure S2. Standard curve of phosphorus standard solutions
